# Advances in theta-burst transcranial magnetic stimulation for auditory comprehension deficits in post-stroke aphasia

**DOI:** 10.3389/fneur.2025.1610016

**Published:** 2025-07-29

**Authors:** Yuling Jing, Haoyang Duan, Wendong Yang, Hong Zhang, Lianxi Dong, Zhenlan Li

**Affiliations:** Department of Rehabilitation Medicine, First Hospital of Jilin University, Changchun, China

**Keywords:** post-stroke aphasia, transcranial magnetic stimulation, auditory comprehension, theta burst stimulation, stroke

## Abstract

Aphasia is a language network disorder caused by organic brain lesions, which severely affects patients' daily communication and interaction. The therapeutic effect of conventional rehabilitation training alone is limited. Currently, Theta Burst Stimulation (TBS) is a novel therapeutic modality of repetitive Transcranial Magnetic Stimulation (rTMS) and is a commonly used patterned rTMS. It appears in the form of burst waves and mimics the natural firing pattern of hippocampal neurons under Theta rhythm. Based on rTMS, TBS embeds a pattern in which three 50 Hz pulse bursts are inserted into a 5 Hz pulse train. This stimulation pattern can induce cortical plasticity in a shorter period of time and is gradually being applied in the treatment of aphasia. Auditory comprehension, as the initial component of language input, involves the reception and storage of linguistic signals, as well as the analysis and integration of lexical semantics. The recovery of this ability plays a prerequisite role in the functional improvement of patients with post-stroke aphasia (PSA). In recent years, research on aphasia has mainly focused on speaking, reading, and writing abilities, with relatively less attention paid to auditory comprehension. Therefore, this article reviews the research progress related to the use of TBS in treating auditory comprehension in aphasia, aiming to provide new ideas and references for the clinical selection of TBS stimulation protocols.

## 1 Introduction

Post-stroke aphasia (PSA) is a language disorder syndrome caused by damage to the language area and related areas of the brain due to organic lesions in the brain, which seriously affects the communication ability and social function of patients (1). Auditory comprehension disorder is one of the common functional impairments in patients with aphasia and also a challenging aspect of aphasia rehabilitation. One of the most important tools for language reception and storage is the auditory channel (2). Therefore, impairment or decline in auditory comprehension can lead to patients' inability to correctly understand others' speech, hindering their capacity to receive and comprehend correct rehabilitation instructions. This, in turn, makes it difficult for them to cooperate with other rehabilitation training programs, thus affecting the overall rehabilitation process after stroke and severely impacting patients' daily life quality. Repetitive transcranial magnetic stimulation (rTMS), a neuroelectrophysiological technique that has developed over the past 20 years, is capable of inducing plastic changes in cortical circuits that last for minutes to hours (3). Studies have shown that rTMS has a promoting effect on the recovery of auditory comprehension function in aphasia after stroke (4). A large-scale retrospective study revealed that safety concerns associated with repetitive transcranial magnetic stimulation (rTMS) primarily involve mild adverse events such as headache and nausea, while the most severe side effect is seizure induction (5). Theta burst stimulation (TBS) is a new therapeutic paradigm for rTMS. In contrast, TBS shows its unique advantages. It not only has a shorter stimulation time, requires fewer stimulation pulses, but also has a relatively lower stimulation intensity. This stimulation method can effectively induce persistent excitatory changes in the cerebral cortex and to a certain extent reduce the risk of side effects (6, 7), thereby garnering significant international scholarly attention. TBS treatment under strict adherence to the indications and contraindications of TBS is relatively safe, with fewer serious adverse reactions such as epileptic seizures. However, it may have mild side effects, such as headache, scalp discomfort, facial muscle twitching, dizziness, nausea, and mild fatigue (8). These are usually temporary. As the treatment progresses, patients will gradually adapt and the side effects will also ease. Once side effects occur, timely symptomatic treatment should be carried out and strict observation should be conducted (9). This article compiles the research progress on the efficacy of TBS in treating auditory and comprehension disorders in patients with aphasia after stroke and reviews it as follows.

## 2 The mechanism of action of TBS

### 2.1 The basic principles of TMS

TMS technology was first proposed by Barker et al. (10). TMS directly stimulates the primary motor cortex of the brain through the skull, which can cause the muscles controlled by this part of the cortex to move. By applying pulsed magnetic field to the central nervous system, the membrane potential of nerve cells can be changed and induced current can be generated, which affects brain metabolism and nerve electrical activity. Current clinical studies have confirmed that rTMS has significantly improved various neurological and psychiatric diseases, especially in various post-stroke after effects, including motor dysfunction (11), swallowing disorder (12), speech (13) and cognitive dysfunction (14), as well as post-stroke depression (15). In recent years, TBS, as a patternized form of rTMS, compared with traditional rTMS, requires patients to remain still for a shorter time during the stimulation process. It has a shorter stimulation time. This time reduction not only improves the treatment efficiency but also increases the patient's comfort and compliance (16). It is widely used in neurological rehabilitation therapy all over the world (17).

### 2.2 Treatment of TBS

**Figure 1:**
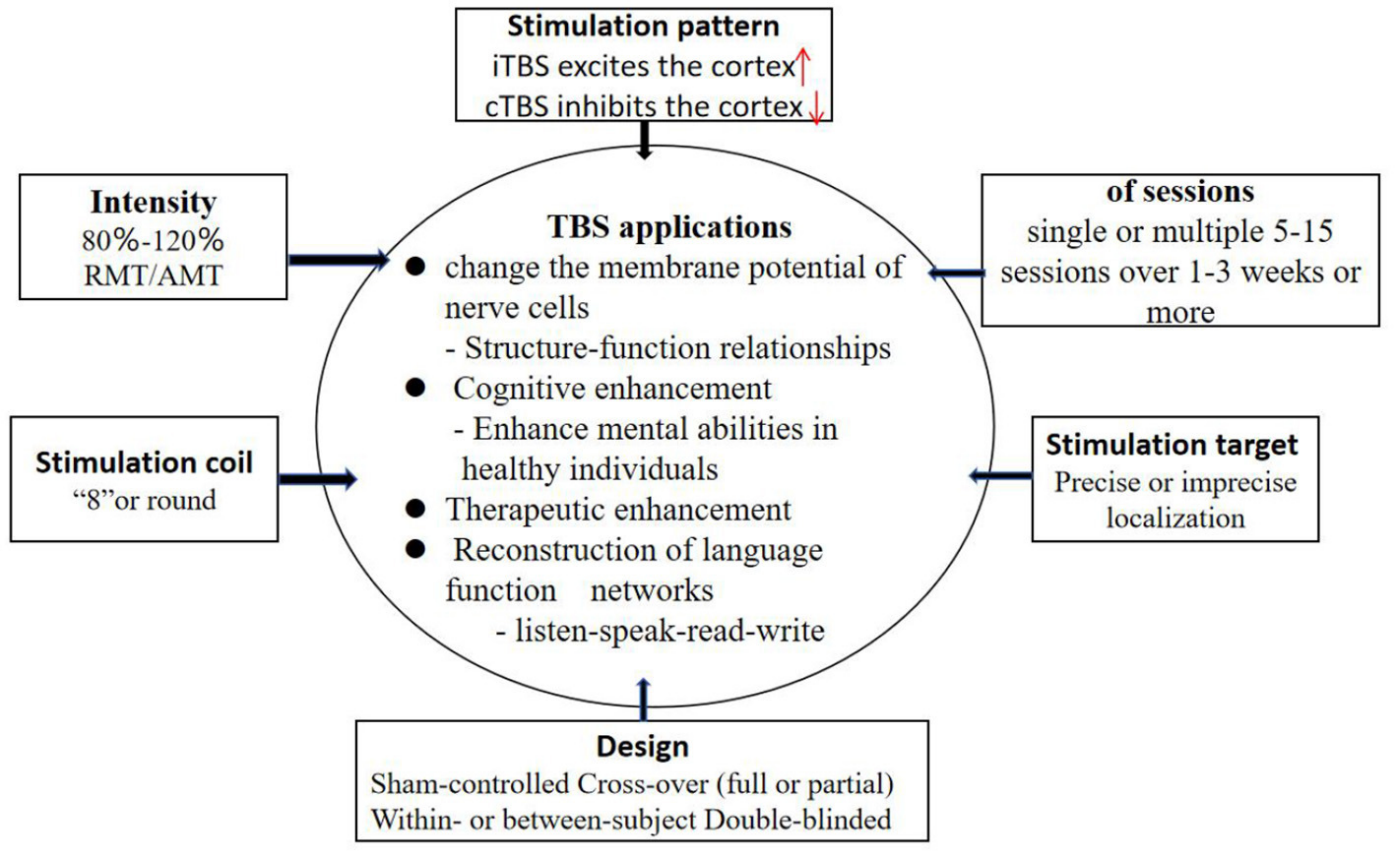
Parameter and design characteristics involving TBS treatment studies.

Theta burst stimulation (TBS), first proposed by Huang et al. (18), is a commonly used model of rTMS, which appears in the form of cluster waves and simulates the discharge pattern in the hippocampus during the processing of information (19). TBS is based on rTMS, which embeds 3 50 Hz pulse clusters into the stimulus mode of 5 Hz pulse. There are two types of stimulation: continuous theta burst stimulation (cTBS) and intermittent theta burst stimulation (iTBS). iTBS stimulates the cerebral cortex continuously for 2 s and pauses for 8 s every 10 s. cTBS is continuously stimulated for 10 s without interval and can inhibit cortical excitability. TBS is characterized by high internal frequency, low stimulus intensity and short duration. The main difference between TBS and traditional rTMS is that the short-term stimulation of TBS (40–190 s) can cause changes in cortical excitability, and this change can continue until at least 20–30 min after the stimulation (20). Studies have shown that TBS can promote sustained changes in cerebral cortex activity in healthy humans that far exceed the duration of traditional TMS (19, 21). Studies have shown that TBS is a more comfortable and effective form of transcranial magnetic stimulation (22, 23). To maximize treatment effectiveness with TBS, standardized methods are needed for methodical selection of TBS parameters (Figure 1).

## 3 Treatment of TBS for auditory comprehension in patients with PSA

According to the theory of “interhemispheric competitive inhibition balance”, healthy subjects rather than normal, the left and right hemispheres of the brain are connected to the corresponding parts through the corpus callosum and inhibits each other to reach a balanced state (24). When one cerebral hemisphere is damaged, the balance is disrupted, and the inhibitory effect of the affected cerebral hemisphere on the healthy side weakens, resulting in increased excitability of the healthy side and enhanced inhibitory effect on the affected side. This interferes with the reactivation of the residual functional network of the dominant hemisphere and is not conducive to the reorganization of neural networks (25). TMS promotes the reconstruction of language function by modulating the imbalance between the two cerebral hemispheres (26). In most individuals, the language-dominant hemisphere is located on the left side. Inhibitory TMS applied to the right cerebral hemisphere can down regulate its excitability, thereby facilitating the recovery of residual language functions on the left side. However, the aforementioned mechanism does not account for the language recovery in all patients with post-stroke aphasia. Therefore, an alternative recovery model known as the “biphasic balance” theory has been proposed (26, 27). When the injury area of the left cerebral hemisphere is relatively small, the remaining language functional area is large and the structural reserve is high, which plays a major compensatory role. According to the “interhemispheric inhibitory balance” model, low-frequency stimulation can be used on the right side or high-frequency stimulation on the left side. Through the intra-hemispheric compensation mechanism, the area around the language-related lesion can be excited. When the area of damage is large and the structural reserve is low, it is necessary to excite the right language mirror area to promote the recovery of language function (28). These mechanisms are not mutually exclusive and may interact during speech recovery in patients with aphasia. The therapeutic effect of TBS on aphasia depends on the degree to which this technique can induce neuroplastic changes during the recombination of language functional areas after stroke (25, 29). Based on the above-mentioned aphasia recovery mechanism, TBS has achieved remarkable results in the recovery of aphasia after stroke in recent years (30, 31). This paper reviews the effect of three stimulation modes on auditory comprehension of PSA patients, and elaborates the improvement of auditory comprehension of PSA patients under three stimulation modes (Table 1).

**Table 1:** Summary of TBS studies in post-stroke aphasia.

**Source**	**Aphasia characteristics**	**Study design**	**Stimulation details**	**Pre-post Tx imaging modality/task**	**Assessment time points**	**Treatment method**	**Outcome**	**Conclusion**
Allendorfer et al. (35)	*N* = 24; Anomic aphasia account for the highest proportion; chronic ischemic stroke	Randomized double-blindsham-controlled trial	Protocol: iTBS group 1 (6): 1W iTBS + 2W sham stimulation (6); iTBS group 2 (6): 2W iTBS + 1 W sham stimulation; iTBS group 3 (6): 3 W iTBS; 600 pulses at 80% AMT; Target: left inferior frontal gyrus; Duration: 5 days/week over 3 weeks	fMRI	Baseline pre TBS, Post, 1 month.	iTBS only	iTBS group: KAAT score about auditory comprehension T1 to T3 (*P* = 0.004) BNT: T1 to T2: (*P* < 0.001), However, T2 to T3 partial decline (*P* = 0.0011); WAB: T1 to T3: (*P* = 0.0011); mini-CAL: T1 to T3 (*P* = 0.0098); VPAT: T1 to T3 (*P* = 0.011); Left ventral visual stream activation fr:T1 to T3 (rho = 0.74, *P* = 0.0058), Sham: Only BNT and VPAT about memory change and no long-term effect	iTBS as an independent treatment can promote the recovery of aphasia after chronic stroke.
Szaflarski et al. (36)	*N* = 27; chronic post-stroke aphasia	A pilot randomized, double-blind, sham-controlled trial	Protocol: iTBS group (20) (G1-3) G1: 1 W iTBS + 2 W sham; G2: 2 W iTBS + 1 W sham; G3: 3 W iTBS; Sham group (7): 3 W sham stimulation 600 pulses at 80% AMT; Target: Left inferior frontal gyrus; Duration: 5 days/week over 3 weeks	fMRI	Baseline, 1 week after treatment, 3-month.	iTBS only	iTBS group: BNT: T1 to T2: (*P* < 0.05). The effect was maintained until 3 month (*P* = 0.056); WAB: T1 to 3 month (*P* = 0.007); Sham group: BNT has improved (possibly due to the practice effect), but there is no long-term effect	The results of this pilot trial support the hypothesis that iTBS applied to the ipsilesional hemisphere can improve aphasia and result in cortical plasticity.
Chou et al. (37)	N = 96; chronic non-fluent aphasia.	RCT	Protocol: iTBS group (32): 600 pulses at 80% RMT; 1HzrTMSgroup (32): 1,200 pulses at 90% RMT; Sham group (32): sham stimulation Target: Brodmann area 45; Duration: 5 days/week over 2 weeks.	No imaging.	Pre-post	iTBS + CIAT; 1 Hz + CIAT	iTBS group: CIAT: (*P* < 0.001) Auditory comprehension: (*P* < 0.001) 1 Hz group: (*P* < 0.001) Sham group: total CCAT score: no significant change.	Compared with low-frequency rTMS, iTBS is more effective in improving the language function of patients with chronic non-fluent aphasia, especially in auditory comprehension.
Kindler et al. (41)	*N* = 18; Anomic aphasia (10), Broca's aphasia (4), others (4)	A randomized, sham-controlled, crossover trial.	Protocol: Each patient received two interventions (cTBS 90% RMT and sham stimulation), with a one-week interval between them; Target: right Brodmann area 45; Duration: 5 days/week over 2 weeks.	MRI: Locate the lesion.	Pre-post	cTBS only	Naming task: (*P* = 0.013) Alertness test (*P* = 0.149) The average post-stroke time of the best responders was 4.7 months, significantly shorter than that of other patients (*P* = 0.009).	cTBS can significantly improve the naming ability of patients with aphasia.
Huang et al. (42)	*N* = 60, PSA for ischemic or hemorrhagic in the left hemisphere.	RCT	Protocol: cTBS group (30): 600 pulses at 90% RMT; Target: Identification of personalized cTBS targets (IFG+STG+SFG) Sham group (30): sham stimulation Duration: 5 days/week over 3 weeks.	rs-fMRI	Baseline, Day 5 (±3 days), Day 21 (±3 days), Day 90 (±7 days).	cTBS+SLT	The article currently only provides the research plan and has not yet reported the specific research results	No conclusion has been reached yet.
Vuksanović et al. (31)	*N* = 1, chronic non-fluent aphasia.	Case report	Protocol: iTBS L-Brodmann area 45 600 pulses at 80% RMT; cTBS R-Brodmann area 45 600 pulses at 80% RMT, Duration: 5 days/week over 3 weeks.	No imaging.	Baseline, 1 week, 2 month.	iTBS + cTBS	BNT: T0-T1-T2: 17-19-21 points Auditory comprehension:word from 49 to 65; BDAE scores improvement was observed.	iTBS and cTBS shows potential to improve language function and speech memory in patients with chronic non-fluent aphasia.

cTBS, continuous theta burst stimulation; iTBS, intermittent theta burst stimulation; RMT, resting motor threshold; AMT, active motor threshold; CIAT, constraint-induced aphasia therapy; fMRI, functional magnetic resonance imaging; KAAT, Kasr Al-Aini Arabic aphasia test; BNT, boston naming test; Mini-CAL, mini-communicative activity log; VPAT, verbal paired associates task; SFT, semantic fluency test; CCAT, concise Chinese aphasia test; RCT, randomized controlled trial; IFG, inferior frontal gyrus; STG, superior temporal gyrus; SFG, superior frontal gyrus; SLT, speech and language therapy; BDAE, boston diagnostic aphasia examination.

### 3.1 Efficacy of iTBS stimulation on the lesion side for auditory comprehension

Functional neuroimaging studies have shown that the key factor for the recovery of language function in patients with aphasia may be the re-recruitment of residual areas of language function in the left hemisphere (32, 33). Although the enhancement of excitability in the mirror area of language function in the right hemisphere during the execution of language tasks may be a compensatory functional recombination, its compensatory effect is not as effective as the re-recruitment of the residual brain area in the left hemisphere (34). Based on this mechanism of aphasia recovery, Allendorfer et al. (35) studied the therapeutic effect of iTBS on the left brain region of 24 patients with aphasia after stroke by using iTBS as an independent treatment. Among them, 6 people received 3 weeks of sham stimulation, and 18 people in iTBS group received 1 week of iTBS + 2 weeks of sham stimulation (6 people). 2 weeks iTBS + 1 week sham stimulation (6 people); The results of the Kasr Al-Aini Arabic Aphasia Test (KAAT) showed significant improvement in the auditory comprehension scores before treatment, immediately after treatment and 1 month after treatment (*P* = 0.004). This suggests that iTBS has a significant positive effect on patients' listening comprehension, not only in the short term, but also over a longer period of time. Moreover, the observed association between iTBS-induced speech improvement and delayed fMRI changes and improvement in aphasia supports the therapeutic and neurorehabilitation potential of iTBS in aphasia recovery after stroke. These findings suggest that iTBS may improve language function by promoting neuroplasticity changes related to auditory processing, visual processing, and motor function. Szaflarski et al. (36) conducted a randomized, double-blind, Sham controlled trial in which iTBS treated 8 patients with aphasia after chronic stroke in the left Broca region. The results of functional magnetic resonance imaging (fMRI) showed that the activation of the language cortex was the largest, and the surrounding white matter was reintegrated. fMRI showed that the language network of patients was significantly transferred to the left hemisphere of the brain. The study believed that the changes in cerebral cortex function of patients after treatment were strongly correlated with the improvement of language function. These results further revealed the excitatory changes of cerebral cortex after iTBS treatment, thus improving the language function of patients with aphasia. This study thus demonstrates once again the potential of neurostimulation as a monotherapy to improve aphasia after chronic stroke and to induce short- and medium-term cortical plasticity in brain networks associated with language function. The research of Chou et al. (37) on non-fluency aphasia after stroke showed that iTBS stimulation of left inferior frontal gyrus was significantly better than low-frequency rTMS stimulation of right inferior frontal gyrus in the total score, matching and listening comprehension of plain Chinese aphasia test, indicating that iTBS can enhance the language recovery of patients with non-fluency aphasia after chronic stroke. ITBS has a higher priority for improving auditory comprehension than low-frequency rTMS.

In conclusion, iTBS stimulates the left hemisphere advantage hemisphere inferior frontal gyrus posterior residual neurons can effectively improve the listening comprehension of the PSA and no adverse reactions occurred in the process of treatment. However, TMS has a high spatial resolution and requires high accuracy in the selection and positioning of stimulation targets. In the above studies, functional imaging was used to treat the most exciting spot in the remaining area as the stimulation target. Neural navigation localization method was used to achieve accurate localization, which may be the key to the remarkable effect of this treatment technique in improving PSA listening comprehension by stimulating the residual neurons in the dominant hemisphere. But the treatment of locating method is high, difficult and costly, may also limit TBS in clinical application.

### 3.2 Efficacy of cTBS stimulation on the non-lesion side for auditory comprehension

RTMS can promote the language rehabilitation of patients with aphasia after stroke, with fewer adverse reactions and high safety, and is strongly recommended in the consensus of clinical experts on aphasia after stroke (38). Similar to low-frequency rTMS, cTBS may have a long-term inhibitory effect on the cerebral cortex. Compared with clinically commonly used rTMS, TBS has the advantages of short stimulation time, small stimulation intensity, long post-action time, and closer to the physiological state of neural activity (6). Functional imaging scans of patients with aphasia after stroke at different periods of onset have been conducted in some studies, and it has been found that the role of the non-dominant hemisphere in aphasia recovery is a dynamic repair process, which varies with the time of stroke onset (29, 39). At present, there are few studies on the intervention of cTBS in stroke patients with aphasia (40). Kindler et al. (41) studied 18 right-handed aphasia patients 0.5 to 57 months after stroke, using the international 10–20 EEG system for localization and cTBS stimulation of the right Broca region, during which the image naming task after listening comprehension and language-independent alertness test were performed. The results showed that compared with false stimulation, patients with cTBS had significantly better performance in auditory comprehension and naming, and the latency period was significantly shortened. The best cTBS treatment effect was in patients with stroke onset of 4.7 months, indicating that cTBS is the best treatment effect in patients with subacute aphasia. However, this study did not carry out functional neural image scanning of the speech functional network, and did not further reveal the changes of the speech functional network before and after cTBS stimulation. Therefore, it is necessary to further explore the mechanism combined with functional images in the future. Huang et al. (42) randomly assigned 60 participants in a 1:1 ratio to the cTBS group and the sham cTBS group. Precise resting state functional magnetic resonance imaging (fMRI) was used to draw personalized language networks for each participant, and personalized targets were designed, including right inferior frontal gyrus, right superior frontal gyrus and right superior temporal gyrus. Each target area was treated with 600 pulses per time, twice a day, a total of 1,200 pulses, and the total pulse number was 3,600 pulses. Participants will receive a three-week cTBS intervention against three personalized targets, combined with SLT therapy. The results showed that the cTBS group showed significantly greater improvement in auditory comprehension and aphasia after 3 weeks of intervention and 1 week after treatment compared to the sham stimulation group, suggesting that the superior temporal and superior frontal gyrus are promising stimulation targets for language recovery after stroke. This also aligns with Mesulam et al. (43), who revealed in the neuroanatomical basis of auditory comprehension ability in patients with primary progressive aphasia that it is closely related to functional areas such as the anterior temporal lobe, but has a limited relationship with the Wernicke area. At present, research on the regions related to auditory comprehension is limited. It is necessary to further apply multi-target TBS stimulation, which provides a new perspective and method for the treatment of aphasia after stroke.

In conclusion, cTBS in the treatment of aphasia is differ, needs further exploration. Studies have found that many factors affect the therapeutic effect of cTBS, such as the duration of onset, the site and size of injury and the type of aphasia, etc. Full consideration of the above influencing factors in the clinical application of patients with aphasia will help to correctly select the treatment plan of TBS and improve the effectiveness of cTBS stimulation in the treatment of patients with aphasia.

### 3.3 Effects of TBS stimulation of bilateral brain and other targets on auditory comprehension

With the deepening of the research on the language central conduction pathway and non-invasive neuroregulation techniques in recent years, iTBS or cTBS alone have achieved reliable efficacy in the treatment of aphasia after stroke. However, the effect of bilateral stimulation with different frequencies on the recovery of aphasia after stroke is worth exploring. However, there are few literatures on the treatment of post-stroke aphasia by iTBS combined with cTBS. Vuksanović et al. (31) applied TBS to the bilateral brain of a patient with non-fluency aphasia who had basal ganglia region damage for 17 months, that is, iTBS stimulated the left language area and cTBS stimulated the right language mirror area. After 5 days of continuous stimulation, it was found that the patients' listening comprehension such as prepositional phrases, vocabulary memory and language fluency were significantly improved. However, this is consistent with the study of Khedr et al. (44) on repetitive transcranial magnetic stimulation for 30 patients with non-fluent aphasia after subacute stroke, which found that auditory comprehension, naming, repetition and fluency were significantly improved after 10 treatments, and such improvement lasted until 2 months after treatment. In addition, patients in the true stimulation group showed a significant increase in cortical excitability in the damaged hemisphere, so bilateral hemispheric TMS may be a viable treatment for non-fluent aphasia, but further multicenter studies are needed to confirm this result. The above study is a single case study, and its accuracy and clinical application value still need to be further explored by expanding the sample size, multi-center, randomized controlled and longer follow-up studies. In addition, TBS combined stimulation of bilateral brain in this study cannot determine the dominant stimulation mode in improving language function, nor can it be determined whether bilateral stimulation mode is superior to single stimulation mode (45, 46).

All the above-mentioned studies stimulated the “classic” target areas of aphasia. However, in recent years, some research suggests that language function is not controlled by a single brain region but is regulated by complex neural networks (47). Therefore, become a research hotspot in recent years, new targets. Zheng et al. (40) conducted cTBS right cerebellum + speech language therapy on 40 patients with aphasia after chronic stroke, and the result was that cTBS stimulation of right cerebellum could increase the effect of SLT on language recovery and regulate the functional connection between right cerebellum and the language processing area of cerebral cortex. At the same time, the effects of cTBS on brain functional connectivity, especially the changes of cerebellar-brain network, will be explored through resting state fMRI data, so as to provide neural mechanism support for the application of cTBS in the treatment of aphasia after stroke and provide a new and feasible treatment plan for the language recovery of aphasia after chronic stroke. In addition, some scholars have reported that iTBS stimulation of cortical motor areas has changes in the brain function of stroke patients with aphasia. Yang et al. (48) recruited 16 patients with PSA and used iTBS to stimulate the left M1 region. The results showed that the functional connections of the semantic network (left frontal lobe, bilateral temporal cortex, left superior limbic gyrus, left cuneus and other regions) were significantly reduced after a single iTBS, but the functional connections of key regions were significantly changed after multiple iTBS, indirectly improving the listening comprehension ability. Therefore, in patients with aphasia, the recombination and functional connection changes of brain semantic networks are closely related to language recovery. iTBS may promote the recovery of language function by regulating the activities of these networks, which also opens up a new perspective for PSA neural rehabilitation.

## 4 Summary

Compared with traditional rTMS, TBS has unique advantages such as short stimulation time, low stimulation intensity and fewer pulses, and is a NIBS technology with great potential (49). Currently, the application of TBS in the treatment of aphasia remains at a relatively early stage. Numerous factors can influence the therapeutic efficacy of TBS for aphasia, including the time since stroke onset, the location of the brain injury, the type of aphasia, the accuracy of the TMS stimulation site, the appropriateness of the treatment protocol, and whether it is combined with SLT (50, 51). Even variables such as age, gender, and genetic factors may impact the clinical outcomes of TBS (30, 33). Therefore, the success or failure of TBS in improving auditory comprehension in post-stroke aphasia may be attributed to both external and/or internal treatment-related factors. It is thus essential to develop individualized treatment plans based on the specific conditions of each aphasia patient in clinical practice.

## References

[1] Wang T Huang X Zhao L Wang Y Zhang S Fu X. A bibliometric analysis of global publication trends on rTMS and aphasia. Medicine. (2023) 102:e33826. 10.1097/MD.0000000000033826 37335693 PMC10194649

[2] Kroczek LOH Gunter TC Rysop AU Friederici AD Hartwigsen G. Contributions of left frontal and temporal cortex to sentence comprehension: evidence from simultaneous TMS-EEG. Cortex. (2019) 115:86–98. 10.1016/j.cortex.2019.01.010 30776735

[3] Sheng R Chen C Chen H Yu P. Repetitive transcranial magnetic stimulation for stroke rehabilitation: insights into the molecular and cellular mechanisms of neuroinflammation. Front Immunol. (2023) 14:1197422. 10.3389/fimmu.2023.1197422 37283739 PMC10239808

[4] Lwi SJ Herron TJ Curran BC Ivanova MV Schendel K Dronkers NF. Auditory comprehension deficits in post-stroke aphasia: neurologic and demographic correlates of outcome and recovery. Front Neurol. (2021) 12:680248. 10.3389/fneur.2021.680248 34456845 PMC8397517

[5] Muller PA Pascual-Leone A Rotenberg A. Safety and tolerability of repetitive transcranial magnetic stimulation in patients with pathologic positive sensory phenomena: a review of literature. Brain Stimul. (2012) 5:320–9.e27. 10.1016/j.brs.2011.05.003 22322098 PMC3656498

[6] Cho YJ Ryu WS Lee H Kim DE Park JW. Which factors affect the severity of dysphagia in lateral medullary infarction? Dysphagia. (2020) 35:414–8. 10.1007/s00455-019-10043-8 31375916

[7] Siddiqi SH Kandala S Hacker CD Trapp NT Leuthardt EC Carter AR. Individualized precision targeting of dorsal attention and default mode networks with rTMS in traumatic brain injury-associated depression. Sci Rep. (2023) 13:4052. 10.1038/s41598-022-21905-x 36906616 PMC10008633

[8] Addicott MA Kinney KR Saldana S Ip EH DeMaioNewton H Bickel WK. A randomized controlled trial of intermittent theta burst stimulation to the medial prefrontal cortex for tobacco use disorder: clinical efficacy and safety. Drug Alcohol Depend. (2024) 258:111278. 10.1016/j.drugalcdep.2024.111278 38579605 PMC11088513

[9] Tang Z Huang J Zhou Y Ren J Duan X Fu X. Efficacy and safety of high-dose TBS on poststroke upper extremity motor impairment: a randomized controlled trial. Stroke. (2024) 55:2212–20. 10.1161/STROKEAHA.124.046597 39016009 PMC11346718

[10] Barker AT Jalinous R Freeston IL. Non-invasive magnetic stimulation of human motor cortex. Lancet. (1985) 325:1106–7. 10.1016/S0140-6736(85)92413-4 2860322

[11] Veldema J Gharabaghi A. Non-invasive brain stimulation for improving gait, balance, and lower limbs motor function in stroke. J Neuroeng Rehabil. (2022) 19:84. 10.1186/s12984-022-01062-y 35922846 PMC9351139

[12] Tai J Hu R Fan S Wu Y Wang T Wu J. Theta-burst transcranial magnetic stimulation for dysphagia patients during recovery stage of stroke: a randomized controlled trial. Eur J Phys Rehabil Med. (2023) 59:543. 10.23736/S1973-9087.23.08023-1 37737051 PMC10664766

[13] Kielar A Patterson D Chou Y. Efficacy of repetitive transcranial magnetic stimulation in treating stroke aphasia: systematic review and meta-analysis. Clin Neurophysiol. (2022) 140:196–227. 10.1016/j.clinph.2022.04.017 35606322

[14] Gao Y Qiu Y Yang Q Tang S Gong J Fan H. Repetitive transcranial magnetic stimulation combined with cognitive training for cognitive function and activities of daily living in patients with post-stroke cognitive impairment: a systematic review and meta-analysis. Ageing Res Rev. (2023) 87:101919. 10.1016/j.arr.2023.101919 37004840

[15] Hordacre B Comacchio K Williams L Hillier S. Repetitive transcranial magnetic stimulation for post-stroke depression: a randomised trial with neurophysiological insight. J Neurol. (2021) 268:1474–84. 10.1007/s00415-020-10315-6 33219421

[16] Hurtado-Puerto AM Nestor K Eldaief M Camprodon JA. Safety considerations for cerebellar theta burst stimulation. Clin Ther. (2020) 42:1169–90. e1. 10.1016/j.clinthera.2020.06.001 32674957

[17] Li KP Wu JJ Zhou ZL Xu DS Zheng MX Hua XY. Noninvasive brain stimulation for neurorehabilitation in post-stroke patients. Brain Sci. (2023) 13:451. 10.3390/brainsci13030451 36979261 PMC10046557

[18] Huang YZ Edwards MJ Rounis E Bhatia KP Rothwell JC. Theta burst stimulation of the human motor cortex. Neuron. (2005) 45:201–6. 10.1016/j.neuron.2004.12.033 15664172

[19] Suppa A Huang YZ Funke K Ridding MC Cheeran B Di Lazzaro V. Ten years of theta burst stimulation in humans: established knowledge, unknowns and prospects. Brain Stimul. (2016) 9:323–35. 10.1016/j.brs.2016.01.006 26947241

[20] Blumberger DM Vila-Rodriguez F Thorpe KE Feffer K Noda Y Giacobbe P. Effectiveness of theta burst versus high-frequency repetitive transcranial magnetic stimulation in patients with depression (THREE-D): a randomised non-inferiority trial. Lancet. (2018) 391:1683–92. 10.1016/S0140-6736(18)30295-2 29726344

[21] Philip NS Barredo J Aiken E Larson V Jones RN Shea MT. Theta-burst transcranial magnetic stimulation for posttraumatic stress disorder. Am J Psychiatry. (2019) 176:939–48. 10.1176/appi.ajp.2019.18101160 31230462 PMC6824981

[22] Yu F Tang X Hu R Liang S Wang W Tian S. The after-effect of accelerated intermittent theta burst stimulation at different session intervals. Front Neurosci. (2020) 14:576. 10.3389/fnins.2020.00576 32670006 PMC7330092

[23] Diekhoff-Krebs S Pool EM Sarfeld AS Rehme AK Eickhoff SB Fink GR. Interindividual differences in motor network connectivity and behavioral response to iTBS in stroke patients. Neuroimage Clin. (2017) 15:559–71. 10.1016/j.nicl.2017.06.006 28652969 PMC5476469

[24] Winhuisen L Thiel A Schumacher B Kessler J Rudolf J Haupt WF. The right inferior frontal gyrus and poststroke aphasia: a follow-up investigation. Stroke. (2007) 38:1286–92. 10.1161/01.STR.0000259632.04324.6c 17322084

[25] Wawrzyniak M Schneider HR Klingbeil J Stockert A Hartwigsen G Weiller C. Resolution of diaschisis contributes to early recovery from post-stroke aphasia. Neuroimage. (2022) 251:119001. 10.1016/j.neuroimage.2022.119001 35172200

[26] Hong Z Zheng H Luo J Yin M Ai Y Deng B. Effects of low-frequency repetitive transcranial magnetic stimulation on language recovery in poststroke survivors with aphasia: an updated meta-analysis. Neurorehabil Neural Repair. (2021) 35:680–91. 10.1177/15459683211011230 34032160

[27] Cheng J Jiang Y Rao T Yang Y Liu Y Zhan Y. Repetitive transcranial magnetic stimulation for post-stroke non-fluent aphasia: a systematic review and meta-analysis of randomized controlled trials. Front Neurol. (2024) 15:1348695. 10.3389/fneur.2024.1348695 38751884 PMC11094331

[28] Thiel A Black SE Rochon EA Lanthier S Hartmann A Chen JL. Non-invasive repeated therapeutic stimulation for aphasia recovery: a multilingual, multicenter aphasia trial. J Stroke Cerebrovasc Dis. (2015) 24:751–8. 10.1016/j.jstrokecerebrovasdis.2014.10.021 25735707

[29] Georgiou A Konstantinou N Phinikettos I Kambanaros M. Neuronavigated theta burst stimulation for chronic aphasia: two exploratory case studies. Clin Linguist Phon. (2019) 33:532–46. 10.1080/02699206.2018.1562496 30676091

[30] Jiang T Wei X Wang M Xu J Xia N Lu M. Theta burst stimulation: what role does it play in stroke rehabilitation? A systematic review of the existing evidence. BMC Neurology. (2024) 24:52. 10.1186/s12883-023-03492-0 38297193 PMC10832248

[31] Vuksanović J Jelić MB Milanović SD Kačar K Konstantinović L Filipović SR. Improvement of language functions in a chronic non-fluent post-stroke aphasic patient following bilateral sequential theta burst magnetic stimulation. Neurocase. (2015) 21:244–50. 10.1080/13554794.2014.890731 24579976

[32] Hartwigsen G Saur D. Neuroimaging of stroke recovery from aphasia–Insights into plasticity of the human language network. Neuroimage. (2019) 190:14–31. 10.1016/j.neuroimage.2017.11.056 29175498

[33] Georgiou AM Kambanaros M. Therapies and challenges in the post-stroke aphasia rehabilitation arena: current and future prospects. Medicina. (2023) 59:1674. 10.3390/medicina59091674 37763793 PMC10537631

[34] Mattioli F Ambrosi C Mascaro L Scarpazza C Pasquali P Frugoni M. Early aphasia rehabilitation is associated with functional reactivation of the left inferior frontal gyrus: a pilot study. Stroke. (2014) 45:545–52. 10.1161/STROKEAHA.113.003192 24309584

[35] Allendorfer JB Nenert R Vannest J Szaflarski JP. A pilot randomized controlled trial of intermittent theta burst stimulation as stand-alone treatment for post-stroke aphasia: effects on language and verbal functional magnetic resonance imaging (fMRI). Med Sci Monit. (2021) 27:e934818–1. 10.12659/MSM.930100 34862359 PMC8653428

[36] Szaflarski JP Nenert R Allendorfer JB Martin AN Amara AW Griffis JC. Intermittent theta burst stimulation (iTBS) for treatment of chronic post-stroke aphasia: results of a pilot randomized, double-blind, sham-controlled trial. Med Sci Monit. (2021) 27:e931468–1. 10.12659/MSM.931468 34183640 PMC8254416

[37] Chou TY Wang JC Lin MY Tsai PY. Low-frequency vs. theta burst transcranial magnetic stimulation for the treatment of chronic non-fluent aphasia in stroke: a proof-of-concept study *Front Aging Neurosci*. (2022) 13:800377. 10.3389/fnagi.2021.800377 35095477 PMC8795082

[38] Lefaucheur JP Aleman A Baeken C Benninger DH Brunelin J Di Lazzaro V. Evidence-based guidelines on the therapeutic use of repetitive transcranial magnetic stimulation (rTMS): an update (2014-2018). Clin Neurophysiol. (2020) 131:474–528. 10.1016/j.clinph.2020.02.003 31901449

[39] Geranmayeh F Brownsett SLE Wise RJS. Task-induced brain activity in aphasic stroke patients: what is driving recovery? Brain. (2014) 137:2632–48. 10.1093/brain/awu163 24974382 PMC4163030

[40] Zheng K Chen M Shen Y Xu X Gao F Huang G. Cerebellar continuous theta burst stimulation for aphasia rehabilitation: study protocol for a randomized controlled trial. Front Aging Neurosci. (2022) 14:909733. 10.3389/fnagi.2022.909733 35721014 PMC9201405

[41] Kindler J Schumacher R Cazzoli D Gutbrod K Koenig M Nyffeler T. Theta burst stimulation over the right Broca's homologue induces improvement of naming in aphasic patients. Stroke. (2012) 43:2175–9. 10.1161/STROKEAHA.111.647503 22581821

[42] Huang J Ren J Xie W Pan R Xu N Liu H. Personalised functional imaging-guided multitarget continuous theta burst stimulation for post-stroke aphasia: study protocol for a randomised controlled trial. BMJ Open. (2024) 14:e081847. 10.1136/bmjopen-2023-081847 38754874 PMC11097845

[43] Mesulam MM Rader BM Sridhar J Nelson MJ Hyun J Rademaker A. Word comprehension in temporal cortex and Wernicke area: a PPA perspective. Neurology. (2019) 92:e224–33. 10.1212/WNL.0000000000006788 30578374 PMC6340389

[44] Khedr EM Abo El-Fetoh N Ali AM El-Hammady DH Khalifa H Atta H. Dual-hemisphere repetitive transcranial magnetic stimulation for rehabilitation of poststroke aphasia: a randomized, double-blind clinical trial. Neurorehabil Neural Repair. (2014) 28:740–50. 10.1177/1545968314521009 24503205

[45] Heiss WD Hartmann A Rubi-Fessen I Anglade C Kracht L Kessler J. Noninvasive brain stimulation for treatment of right-and left-handed poststroke aphasics. Cerebrovasc Dis. (2013) 36:363–72. 10.1159/000355499 24217362

[46] Nissim NR Moberg PJ Hamilton RH. Efficacy of noninvasive brain stimulation (tDCS or TMS) paired with language therapy in the treatment of primary progressive aphasia: an exploratory meta-analysis. Brain Sci. (2020) 10:597. 10.3390/brainsci10090597 32872344 PMC7563447

[47] Siebner HR Funke K Aberra AS Antal A Bestmann S Chen R. Transcranial magnetic stimulation of the brain: What is stimulated?–a consensus and critical position paper. Clin Neurophysiol. (2022) 140:59–97. 10.1016/j.clinph.2022.04.022 35738037 PMC9753778

[48] Yang Q Xu S Chen M Deng P Zhuang R Sun Z. Effects of the left M1 iTBS on brain semantic network plasticity in patients with post-stroke aphasia: a preliminary study. J Integr Neurosci. (2023) 22:24. 10.31083/j.jin2201024 36722227

[49] Cole E O'Sullivan SJ Tik M Williams NR. Accelerated theta burst stimulation: safety, efficacy, and future advancements. Biol Psychiatry. (2024) 95:523–35. 10.1016/j.biopsych.2023.12.004 38383091 PMC10952126

[50] Bai G Jiang L Ma W Meng P Li J Wang Y. Effect of low-frequency rTMS and intensive speech therapy treatment on patients with nonfluent aphasia after stroke. Neurologist. (2021) 26:6–9. 10.1097/NRL.0000000000000303 33394904

[51] Latorre A Rocchi L Berardelli A Bhatia KP Rothwell JC. The use of transcranial magnetic stimulation as a treatment for movement disorders: a critical review. Mov Disord. (2019) 34:769–82. 10.1002/mds.27705 31034682

